# Chicago Medical Society

**Published:** 1879-09

**Authors:** R. Park


### Society Reports.

### ARTICLE XII.

CHICAGO MEDICAL SOCIETY. (Reported by R. Park, M.D.) Regular meeting July 7th. Dr. E. L. Holmes, chairman *pro tem.*

Dr. A. R. Jackson read a paper on "Lacerations of the Cervix Uteri," which will be found in the last number of the JOURNAL.

In the discussion which followed, DR. DUDLEY said he wished to correct any impression that Dr. Gardner was entitled to claim priority of discovery of all that Dr. Emmet had called attention to. The former had recognized the fact that laceration existed in many of these cases, but had not noticed, or at least had not laid any stress on the fact, that by a proper approximation of the edges of the rent the parts were reduced to their proper position, and much of the enlargement disappeared.

DR. FITCH wanted to be understood as recognizing the frequent occurrence of the lesion, and its importance as a factor in much of the suffering which women have to bear; but he thought, after all, that it was not the extent of the laceration, be it slight or great, that should determine the advisability of Emmet's operation, but the effect of the lesion upon the patient, judging from the consequent pain, lassitude, sterility, etc., of which she might complain. He had seen cases where extensive laceration had taken place, while the patient suffered very little, and he doubted if the surgeon should, in such a case, operate. That trachelorrhaphy does not prevent laceration again in subsequent labor, he learned by experience in two cases. With the essayist, he insisted on the necessity for careful preparatory treatment; but he feared that weekly puncture for purposes of depletion would lead to a cicatricial contraction, which would complicate the results of an operation. He did not believe there was any need to resort to anæsthesia during the operation, nor to the use of the catheter during the few days after it; but looked forward to the time when we should operate in our offices and then send our patients about their various duties.

DR. TILLEY enquired whether there was no prophylaxis during and subsequent to labor which should be insisted upon.

DR. INGALS was surprised that in examinations the unaided finger and the bivalve or round speculum should reveal so little, while the Sim's speculum or a perineal retractor revealed so much.

DR. BARTLETT related a case of Dr. Mary Thompson's. A woman suffering from cervical laceration became the subject of epileptic seizures, and finally had in one day six or eight of these attacks. The doctor operated in the usual way, and the patient had now had but one fit in three months. Dr. Bartlett also referred to a paper which he had read before the Society of Physicians and Surgeons, "On the Anatomy of the Cervix Uteri, Before, During and After Labor," and called attention to the facts therein pointed out as having a new interest in connection with the subject under discussion. Stolby, Cazeaux, and the majority of more recent writers, state that up to a few weeks before confinement the cervix remains entirely undeveloped, and that the last joint of a finger inserted along the cavity of the neck may reach the os internum; that during the progress of labor the ring of the cervix is obliterated, so that the uterus and vagina form a continuous canal; and that after labor the strongly "pursed" orifice through which the fingers pass in entering the cavity of the womb is the os externum.

In the paper referred to, Dr. Bartlett had stated that before labor the cervix had greatly expanded, so that even that portion of it below the vaginal attachment measured several inches in width, that the contracted portion really felt by Cazeaux *below* the vaginal attachment, and yet located in his imagination—by a singular perversion of conception—*above* it, was not, as he supposed, the os internum, but that segment of the cervix which, at the time of examination, was the line of demarcation between the expanded and the yet unexpanded portion of the neck. That the infra-vaginal portion of the cervix is not "obliterated" at the time of the passage of the child, but remains as a narrow band jutting into the parturient canal. And finally, he had stated that after labor the firmly contracted orifice generally alluded to as the os, is the os internum, while the infra-vaginal portion of the cervix floats in the vagina as a flabby ring.

It was, therefore, the fact of the existence of this ring which he wished to introduce in this connection. For while no one is able to conceive of those conditions which are now recognized as cervical lacerations as being the outcome of the slight, superficial, purse-mouth flutings which may be sometimes felt in the margin of the os after labor, yet, given an actual, substantial, fleshy ring, through which is to be propelled a fœtus, it is easy to conceive of the cause, the time, the anatomy, the means of diagnosis, and even the plan of treatment of those injuries of the uterine neck which are just now of such paramount interest to the gynæcologist.

DR. WICKERSHAM thought that the general practitioner should learn, from the paper and its discussion, that in all cases of parturition he should avoid meddlesome midwifery.

In closing the discussion, DR. JACKSON said he thought Dr. Fitch's suggestions with regard to the feasibility of operating were admirable. He also allowed that "meddlesome midwifery" might be and was the frequent cause of much future trouble, as well in the direction of the subject of his paper as in other ways. He would insist on the general rule that every accoucheur should examine his patients four to six weeks after confinement, in order to detect laceration of the cervix uteri as of the perineum.

After this discussion had ceased, DR. C. FENGER gave an elaborate demonstration of the pathology of embolism, illustrating his remarks by crayon sketches, morbid specimens and sections under the microscope. He divided emboli into innocent and noxious, and classified their ultimate effects thus: I, None; the resulting ischæmia being compensated for by collateral circulation. II, Gangrene; which usually took place only in the extremities. III, Hæmorrhage; for the most part occurring in parenchymatous organs. IV, Abscess; *e. g.*, pyæmic abscesses. Innocent emboli might cause II and III, but never IV; noxious emboli, III and IV. He described the process of fatty degeneration of embolic infarctions and the formation of abscess from the same.

Specimens were exhibited from two cases: one of embolism and abscess of the liver; the other of septic endocarditis with infarctions in the brain, spleen, liver and kidneys.

